# Prevalence of Sleep Problems Among Chinese Medical Students: A Systematic Review and Meta-Analysis

**DOI:** 10.3389/fpsyt.2022.753419

**Published:** 2022-03-09

**Authors:** Yuanlong Sun, Huiying Wang, Tao Jin, Fei Qiu, Xiaolong Wang

**Affiliations:** ^1^Department of Internal Medicine, Shuguang Clinical Medical College, Shanghai University of Traditional Chinese Medicine, Shanghai, China; ^2^Cardiovascular Department, Cardiovascular Research Institute of Traditional Chinese Medicine, Shuguang Hospital Affiliated With Shanghai University of Traditional Chinese Medicine, Shanghai, China

**Keywords:** Chinese medical students, prevalence, cross-sectional studies, meta-analysis, sleep problems

## Abstract

**Background:**

Sleep is a necessary physiological process, which is closely related to cognitive function, emotion, memory, endocrine balance, and immunity. The prevalence of sleep problems continues to rise in Chinese medical students, which has a potential influence on living and work.

**Objective:**

This study aimed to observe the prevalence of sleep problems among medical students in China.

**Method:**

The included cross-sectional studies on the prevalence of sleep problems of medical students in China were retrieved from PubMed, Embase, the Cochrane Database of Systematic Reviews, CNKI, and Wanfang database. An 11-item checklist recommended by the Agency for Healthcare Research and Quality was adopted to evaluate the methodological quality of the included studies. Software Stata 12.0, SPSS 26.0, and R were used to analyze the data. Registration: PROSPERO, CRD 42021237303.

**Result:**

The prevalence of sleep problems among Chinese medical students was 27.38%. The subgroup analysis showed significant differences in the prevalence of sleep problems among different regions, educational backgrounds, grades, and University types. The region, latitude, and gross domestic product (GDP) were significant heterogeneous sources of sleep problems. The prevalence is positively correlated with latitude and negatively correlated with GDP per capita. Regular screening and appropriate intervention are recommended for these mental health problems.

**Systematic Review Registration:**

https://www.crd.york.ac.uk/prospero/display_record.php?ID=CRD42021237303, identifier: CRD42021237303.

## Introduction

Sleep problems including insomnia, hypersomnia, daytime sleep, and so forth (1, 2) not only reflects the low efficiency of rest but also has significant negative effects on an individual's daily ability, including impaired work performance, low physical and work efficiency, reduced quality of life, and increased risk of secondary mental illness (3). Sleep problems may also be the cause and symptom of mental disorders and somatic symptoms (4–7). Sleep disturbance may be a comorbid factor, etiology, or symptom of depression (8, 9), and increases the risk of depression (10, 11). Insomnia may increase the risk of mental illness, including depression and anxiety (12, 13), lead to physical damage, lower quality of life, high risk of suicidal ideation (14, 15), and hospitalization tendency (16).

In medical schools, medical students are trained into competent and compassionate doctors to improve medical knowledge, help patients, and promote public health (17). Compared with other majors, a higher incidence of sleep problems in medical students (18) may be due to the vast range of academic courses, the high level of clinical work intensity (19, 20), the overnight and on-call shifts during the internship (21, 22) for medical students to achieve the necessary professional knowledge and skills (16, 23). Regular screening and interventions for sleep problems may help control the risk of developing psychological symptoms in Chinese medical students.

At present, many reports on sleep problems have been published, but few systematic reviews or meta-analysis of that in Chinese medical students exists. In addition, the assessment of sleep problems among students of different ages, educational backgrounds, and majors, such as the difference between medical students majoring in traditional Chinese medicine (TCM) and Western medicine, is lacking. Hence, the problem of sleep problems in Chinese medical education remains unclear.

This review aimed to comprehensively assess the prevalence of sleep problems of Chinese medical students, and ascertain the impact of different characteristics of students might have a different effect on the prevalence to discover a pattern of sleep problems of medical students based on available evidence and provide professional guidance for medical students to adapt to the college life.

## Methods

This review was conducted following the Preferred Reporting Items for Systematic Reviews and Meta-Analyses Protocols 2015 statement (24, 25).

### Search Strategy

Five computer databases (PubMed, Embase, the Cochrane Database of Systematic Reviews, CNKI, and Wanfang) were searched. The search time was from the completion of the database to June 2021. No restrictions were imposed on language and publishing status. We searched databases with search formulas and keywords included in these formulas. This search strategy aimed to collect researches on sleep problems among Chinese medical students.

The search formula was as follows:

#1: ‘Sleep problems' OR ‘Poor sleep quality' OR ‘Low sleep quality' OR ‘Sleep disorders' OR ‘Sleep disturbance' OR ‘Insomnia' OR ‘Agrypnia' OR ‘Hypersomnia' OR ‘Hypersomnolence' OR ‘Parasomnia' OR ‘Dyssomnia' OR ‘Sleep deprivation' OR ‘Somnipathy' OR ‘Obstructive sleep apnea' OR ‘OSA' OR ‘Restless legs syndrome' OR ‘RLS' OR ‘behaviorally induced insufficient sleep syndrome' OR ‘BIISS' OR ‘circadian rhythm disorder' OR ‘nightmare' OR ‘Day time sleep'

#2: ‘Chinese medical students' OR ‘medical students' AND ‘China'

#3: #1 AND #2

### Study Selection

This review investigated studies include researches of prevalence of sleep problems among Chinese medical students. Eligible studies should meet the inclusion criteria: (1) Cross-sectional studies, (2) Date of publication before 18 June, 2021, (3) Chinese medical students in universities are assessed as the target population, (4) Reported the prevalence of sleep problems of Chinese medical students or provided sufficient data for calculating the prevalence, (5) Validated measurements were utilized as assessing tool for sleep problems, such as the Pittsburgh Sleep Quality Index (PSQI) for sleep quality assessment, eight-item Epworth Sleepiness Scale (ESS) for excessive daytime sleepiness, Athens Insomnia Scale (AIS) and Diagnostic and Statistical Manual of Mental Disorders (DSM) for insomnia and short sleep. The exclusion criteria are: (1) Intervention studies, case reports and cases comments, (2) Lack of data to calculate the prevalence of sleep problems, (3) Abstracts with no full text, (4) Studies that reported results include Chinese medical students, but without a subgroup analysis of that, (5) Studies investigating data under special circumstance, such as observation during offshore internship.

### Study Registration

This systematic review was conducted following the registration on the prospective register of systematic reviews (PROSPERO, CRD42021237303). The filtering of studies was not performed before registration.

### Study Filtering and Data Extraction

**Figure 1:**
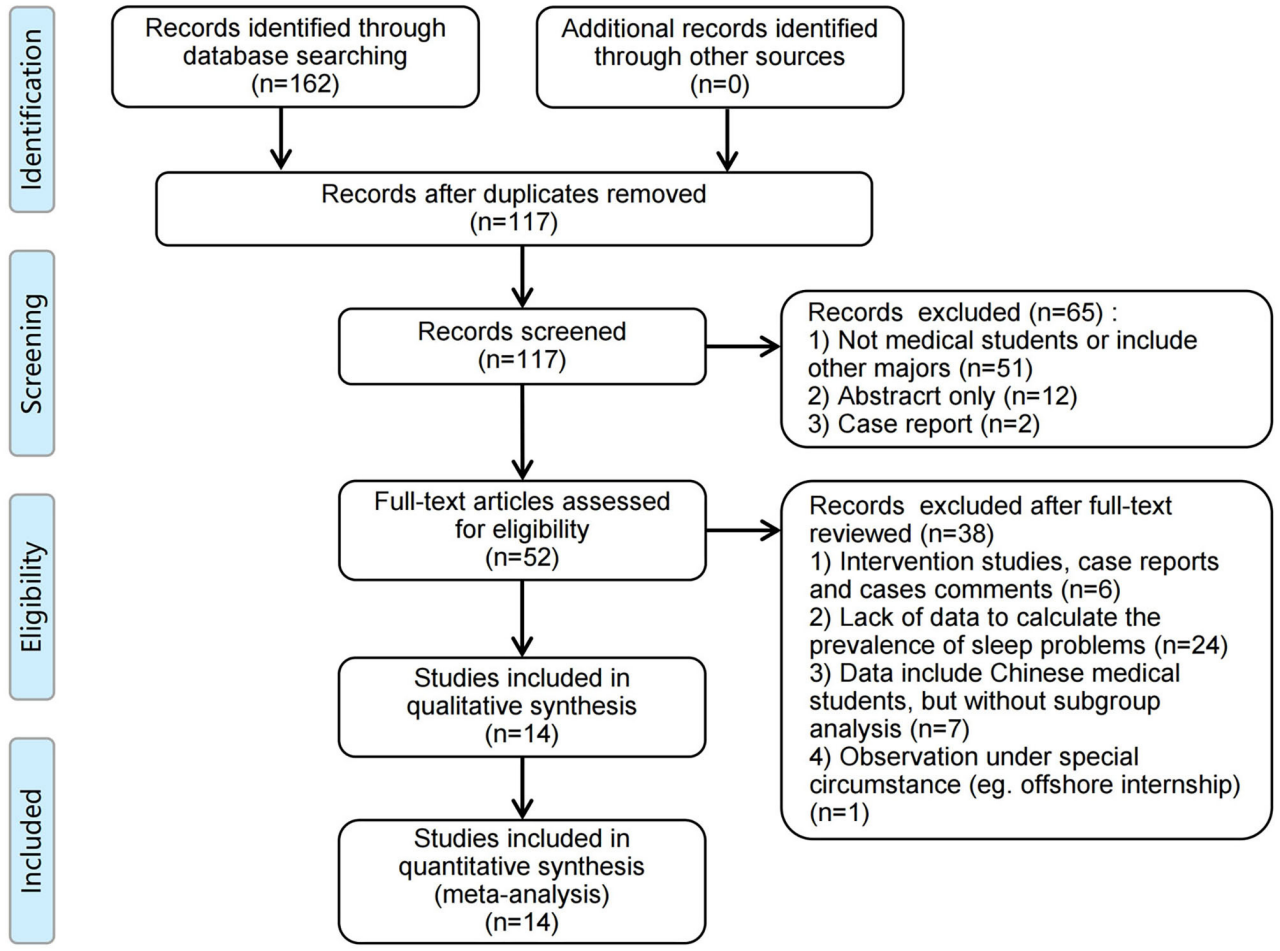
PRISMA flow chart for the article filtering process.

Two independent reviewers performed the full-text reviewing and data extraction process, and a third researcher checked the consistency. The disputes in the process were resolved through discussion with methodology experts (Figure 1). After the study was included, two reviewers independently extracted the data, including (1) the basic information of the study: author, measurements, publication year, study design, sample size, and proportion of participants; (2) information of participants: region, average age, grade, gender, education background, location of universities, and so forth; (3) outcome data: total prevalence and prevalence of different subgroups; (4) data of the latitude, gross domestic product (GDP) and GDP per capita of the cities where the universities are. The latitude and GDP data were collected from city data website (https://www.swguancha.com/).

### Quality Evaluation

The Agency for Healthcare Research and Quality (AHRQ) with an 11-item checklist was applied to access the methodological quality and risk of bias of the included studies. An item was scored “0” for the answer is “NO” or “UNCLEAR” and the item was scored “1” when answered “YES.” Then The study quality was evaluated with three standards: low quality = 0–3, moderate-quality = 4–7, and high quality = 8–11 (26).

### Data Synthesis and Analysis

Two independent researchers collected and input the data into Stata 12.0 software (www.stata.com) for analysis, including meta-analysis, subgroup analysis, and meta-regression. Sleep problems was used as the main outcome. The extracted data were used to calculate the prevalence rate and standard errors in prevalence. Data of prevalence met the condition: *n* × *p* >5 and *n* × (1 – *p*) >5. The formula for calculating the standard error (SE) was as follows:


$$\begin{array}{l} \text{SE}=\sqrt{\frac{\text{p}\times \text{(1 - p)}}{n}} \end{array}$$

where *p* is the prevalence, and *n* denotes the number of patients. This formula was applied to both total prevalence and prevalence between subgroups.

The prevalence of included study was collected or calculated. The combined effect was obtained by the inverse-variance analysis, and the weighted mean was calculated using the SE. The *P*-value and 95% confidence interval (16) were statistically analyzed. The Cochran *Q* test and the Higgins *I*^2^ test were performed to evaluate the heterogeneity across studies (27). The fixed-effect models were used for low between-study heterogeneity (*P* > 0.05 and *I*^2^ <50%). The random-effects models were used for significant heterogeneity (*P* <0.05 and *I*^2^ > 50%). Jackknife analysis will be performed as sensitivity analysis to assess the robustness of the synthesized results. The following variables were used in subgroup analysis: sex, education background, grade, University type, region. The following variables were used in meta-regression: sex, education background, grade, University type, region, publication year, measurements, average age, sample size, latitude, GDP and GDP per capita. The results were assessed with forest plots. Publication bias was conducted with Egger's and Begg's tests, and the results were evaluated with bubble plots.

The statistical significance of prevalence and other continuous variables were analyzed with the Spearman correlation coefficient (CC). Statistical Product and Service Solutions (SPSS) version 26.0 was used to do the correlation analysis.

## Results

One hundred and sixty two related studies were retrieved, and 14 of them were included in this analysis (Figure 1). All included studies were cross-sectional design, and 21,848 Chinese medical undergraduate and graduate students in total were involved.

### General Information

Medical students involved in the analysis were all from China. Among the included studies, five studies were conducted in South China and five studies in North China. Two concealed the geographical area of the study object due to confidentiality agreement. Two studies were published in English, and 10 studies were published in Chinese. All included studies used the lists of validated self-report questionnaires as a measurement to evaluate students. PSQI, eight-item Epworth Sleepiness Scale (28), and Diagnostic and Statistical Manual of Mental Disorders (DSM) were used for assessing sleep problems (Table 1).

**Table 1:** Characteristics of the included studies.

**References**	**Study design**	**City**	**Latitude**	**Age, *x* ±SD and/or range**	**Grade**	**Sample size**	**Male/Female**	**Response rate, %**	**Method (cut off point)**	**AHRQ**
Feng et al. (29)	Cross-sectional	/	/	20.17 ± 1.50	1–4	480	254/226	93.2	PSQI (>7)	7
Jiang et al. (30)	Cross-sectional	/	/	21.04 ± 1.84	1–5	473	140/333	95	PSQI (>7)	5
Lai (31)	Cross-sectional	Nanchang	28.67	20.30 ± 1.31	1–3	581	275/306	96.83	PSQI (>7)	7
Li et al. (32)	Cross-sectional	Changchun	43.88	21.54 ± 1.98	1–5	648	226/422	88.52	PSQI (NA)	5
Li et al. (33)	Cross-sectional	Guangzhou	22.93	/	1–3	951	337/614	92.15	PSQI (>7)	5
Mou et al. (34)	Cross-sectional	Changchun	43.88	/	1–4	207	53/154	86.25	PSQI (>=7)	6
Qi and Zhai (35)	Cross-sectional	/	/	17–22	1–4	960	663/297	/	DSM-III (NA)	6
Shen et al. (36)	Cross-sectional	Yiyang	28.57	17–22	/	4,882	537/4,345	97.56	ESS (>=11)	7
Wang et al. (19)	Cross-sectional	Huhehaote	40.81	/	1–5	6,085	1,660/4,425	/	PSQI (>7)	9
Wang et al. (23) and Wang et al. (37)	Cross-sectional	Wuhu	31.33	18.80 ± 1.18	1–3	3,738	2,186/1,552	98.37	PSQI (>7)	7
Xiao et al. (38)	Cross-sectional	/	/	23.16 ± 2.33	1–8	902	401/501	90.2	PSQI (>7)	9
Yang et al. (39)	Cross-sectional	/	/	20.71 ± 1.23	1–4	584	299/285	97.01	PSQI (>=8.5)	6
Yang et al. (40)	Cross-sectional	Lasa	29.66	/	/	220	96/124	89.43	PSQI (>7)	6
Jie et al. (41)	Cross-sectional	Hefei	31.86	21.16 ± 1.44	1–5	1,137	542/595	98.02	PSQI (>7)	9

*PSQI, Pittsburgh Sleep Quality Index; ESS, eight-item Epworth Sleepiness Scale; DSM, Diagnostic and Statistical Manual of Mental Disorders; AHRQ, Agency for Healthcare Research and Quality (quality score)*.

### Quality Evaluation

The methodology and quality of all included studies were evaluated through AHRQ. The quality scores are shown in the last column of Table 1. The meta-analysis included 11 moderate-quality studies and 3 high-quality studies (Supplementary Table 1).

### Results of the Meta-Analysis

**Figure 2:**
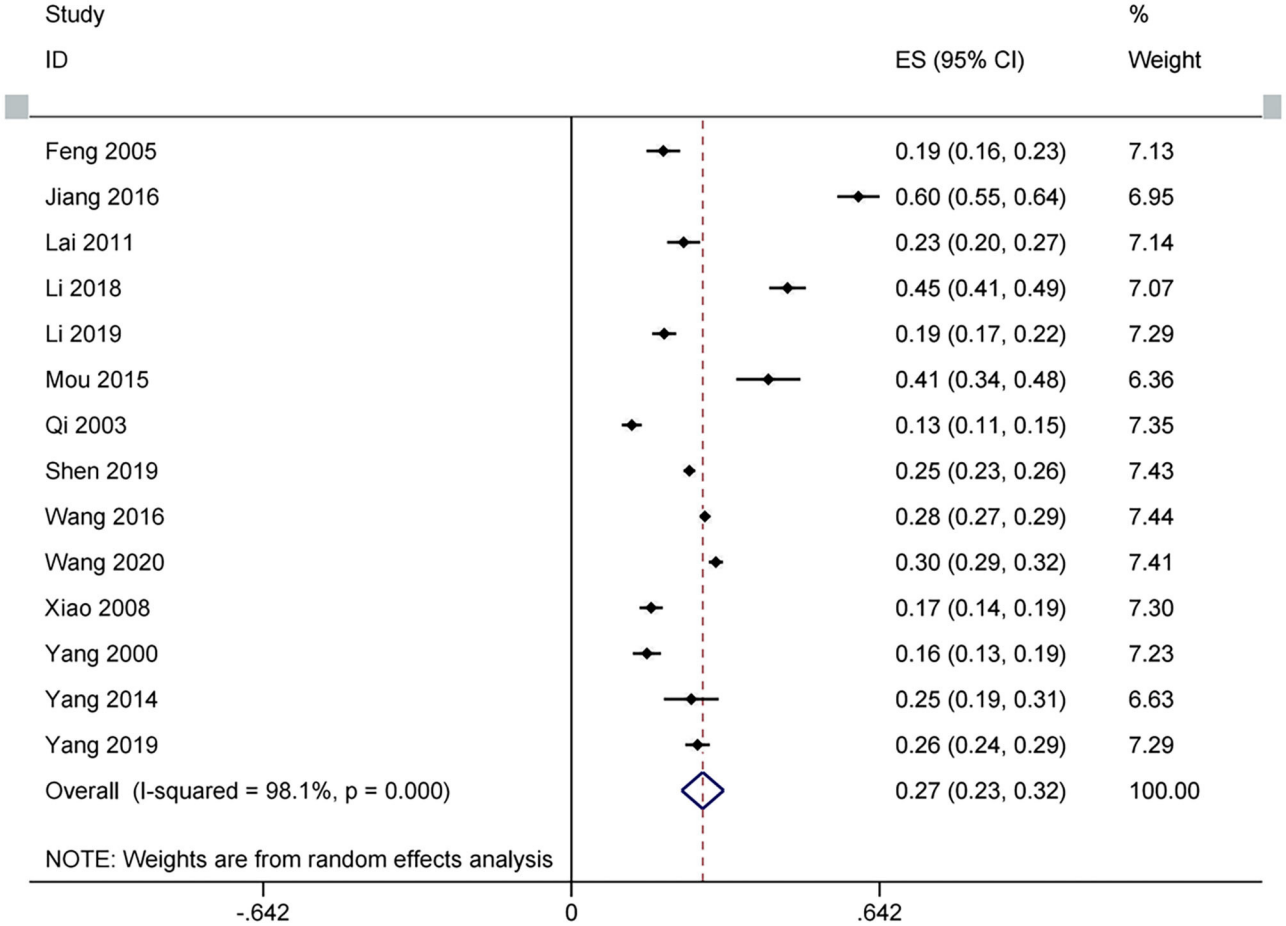
Forest plots of sleep problems of Chinese medical students based on prevalence.

**Figure 3:**
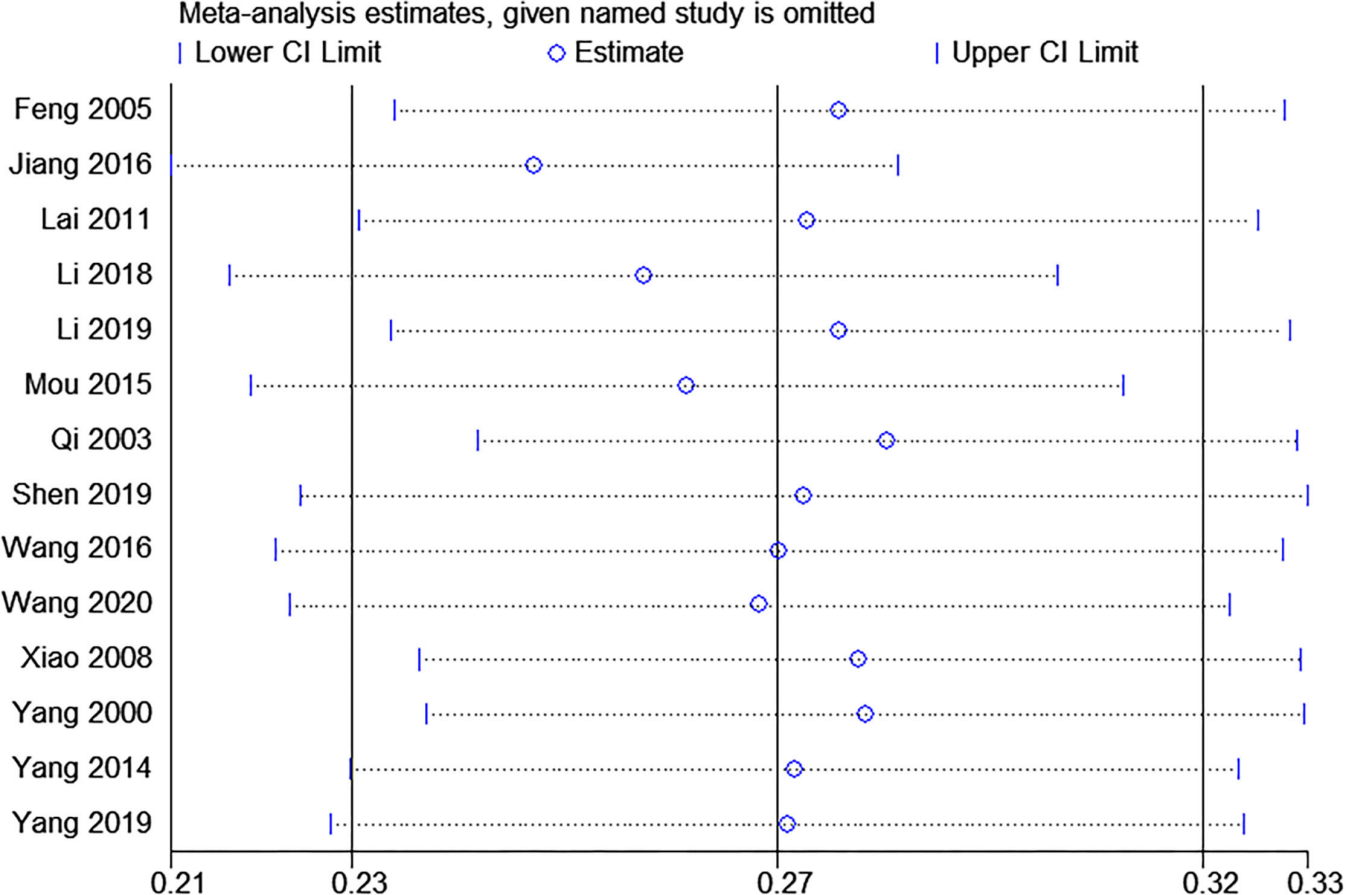
Sensitivity analysis with jackknife analysis.

Fourteen studies were included (19, 29–41). Random effect was used as between-study heterogeneity is significant (tau^2^ = 0.01, Chi^2^ = 686.42, I^2^ = 98%, *P* <0.00001). As shown in Figure 2, the prevalence of sleep problems in Chinese medical students is 27.38% (95% CI: 0.23–0.32, *P* <0.00001), with a range from 12.6 to 59.83%. Sensitivity analysis revealed that no study influenced the synthesized results by more than 2% (Figure 3).

### Results of Subgroup Analysis

The subgroup analysis on the prevalence of sleep problems in Chinese medical students showed that the differences in the prevalence of sleep problems in male (23.36%, 95% CI: 0.19–0.28, *P* <0.00001) and female (23.71%, 95% CI: 0.18–0.30, *P* <0.00001) medical students were not statistically significant (*P* = 0.93, *I*^2^ = 0%). The prevalence in medical students in north China (33.96%, 95% CI: 0.28–0.40, *P* <0.00001) was higher than that in south China (24.89%, 95% CI: 0.20–0.29, *P* <0.00001); the difference between subgroups was significant (*P* = 0.01, *I*^2^ = 84.0%). The prevalence in undergraduate students (28.61%, 95% CI: 0.23–0.34, *P* <0.00001) was higher than that in graduate students (12.83%, 95% CI: 0.10–0.16, *P* <0.00001); the difference between subgroups was significant (*P* <0.00001, *I*^2^ = 95.5%). Among all undergraduate students, the prevalence was slightly higher in Grade 1 (30.88%, 95% CI: 0.17–0.45) and increased with the grade since Grade 2 (Grade 2, 28.35%, 95% CI: 0.11–0.46; Grade 3, 30.84%, 95% CI: 0.14–0.48; Grade 40.87%, 95% CI: 0.15–0.67; Grade 5, 60%, 95% CI: 0.46–0.74); the difference between subgroups was significant (*P* = 0.01, *I*^2^ = 69%). When stratified by the type of University into Western medicine and TCM, the medical students in Western medicine universities (28.02%, 95% CI: 0.23–0.33, *P* <0.00001) had a higher prevalence of sleep problems compared with students in TCM universities (19.35%, 95% CI: 0.17–0.22, *P* <0.00001); the difference between subgroups was significant (*P* = 0.001, *I*^2^ = 90.40%) (Table 2).

**Table 2:** Results of the subgroup analysis of sleep problems.

**Subgroups**	**Number**	**Prevalence (95%CI)**	**Between-study heterogeneity**		**Z**	**Over all *P* Value**	**Between-group difference**		
			***P* Value**	**I^**2**^ (%)**			***P* Value**	**Chi^**2**^**	**I^**2**^(%)**
Gender							0.93	0.01	0
Male	10	0.23 (0.19, 0.28)	<0.00001	94	9.93	<0.00001			
Female	10	0.24 (0.18, 0.30)	<0.00001	98	12.19	<0.00001			
Region							0.02	5.49	82
Northern	4	0.34 (0.28, 0.40)	<0.00001	97	10.95	<0.00001			
Southern	5	0.25 (0.20, 0.29)	<0.00001	93	10.75	<0.00001			
Education							<0.00001	22.01	96
Undergraduate	11	0.29 (0.23, 0.34)	<0.00001	98	9.73	<0.00001			
Postgraduate	1	0.13 (0.10, 0.16)	/	/	7.87	<0.00001			
Grade							0.01	12.90	69
1	4	0.31 (0.17, 0.45)	<0.00001	95	4.42	<0.00001			
2	4	0.28 (0.11, 0.46)	<0.00001	97	3.21	0.001			
3	4	0.31 (0.14, 0.48)	<0.00001	96	3.61	0.0003			
4	2	0.41 (0.15, 0.67)	<0.00001	93	3.08	0.0002			
5	1	0.60 (0.46, 0.74)	/	/	8.66	<0.00001			
University type							0.001	10.43	90
Western	13	0.28 (0.23, 0.33)	<0.00001	98	11.87	<0.00001			
Traditional	1	0.19 (0.17, 0.22)	/	/	12.25	<0.00001			

### Results of Meta-Regression

The meta-regression was performed for sensitivity analysis. The results showed that region (adj *R*^2^ = 51.15%, *P* = 0.020), latitude (adj *R*^2^ = 73.49%, *P* = 0.003) and GDP (adj *R*^2^ = 56.07%, *P* = 0.037) per capita were main sources of heterogeneity of sleep problems, but not gender (*P* = 0.992), average age (*P* = 0.952), education background (*P* = 0.314), grade (*P* = 0.272), University type (*P* = 0.532), publication year (*P* = 0.053), measurements (*P* = 0.461), sample size (*P* = 0.822) and GDP (*P* = 0.253) (Table 3).

**Table 3:** Results of meta-regression analysis.

**Variables**	**Number of studies**	***P*-Value**	**Tau^**2**^**	**I^**2**^ (%)**	**Adj R^**2**^ (%)**
Gender	20	0.992	0.8841	97.10	−5.98
Average age	8	0.952	0.0273	98.60	−17.06
Region	9	0.020	0.0031	95.27	51.15
Education	12	0.314	0.8392	98.43	1.06
Grade	15	0.272	0.0356	95.62	1.98
University type	14	0.532	0.0171	98.19	−4.98
Publication year	14	0.053	0.0127	97.32	22.35
Measurements	14	0.461	0.0169	98.17	−3.62
Sample size	14	0.822	0.0176	98.13	−8.28
Latitude	9	0.003	0.0017	93.56	73.49
GDP (2019)	7	0.253	0.0072	95.38	10.53
GDP per capita (2019)	7	0.037	0.0035	93.64	56.07

*GDP, Gross Domestic Product*.

### Correlation Analysis

We analyzed the correlations between prevalence, and various continuous variables (Supplementary Table 3). The prevalence showed positive correlations with publication year (*r* = 0.630, *P* = 0.016), latitude (*r* = 0.929, *P* <0.001), and negative correlations with GDP per capita (*r* = −0.919, *P* = 0.003). Negative correlations were found between latitude and GDP per capita (*r* = −0.891, *P* = 0.007).

### Publication Bias

**Figure 4:**
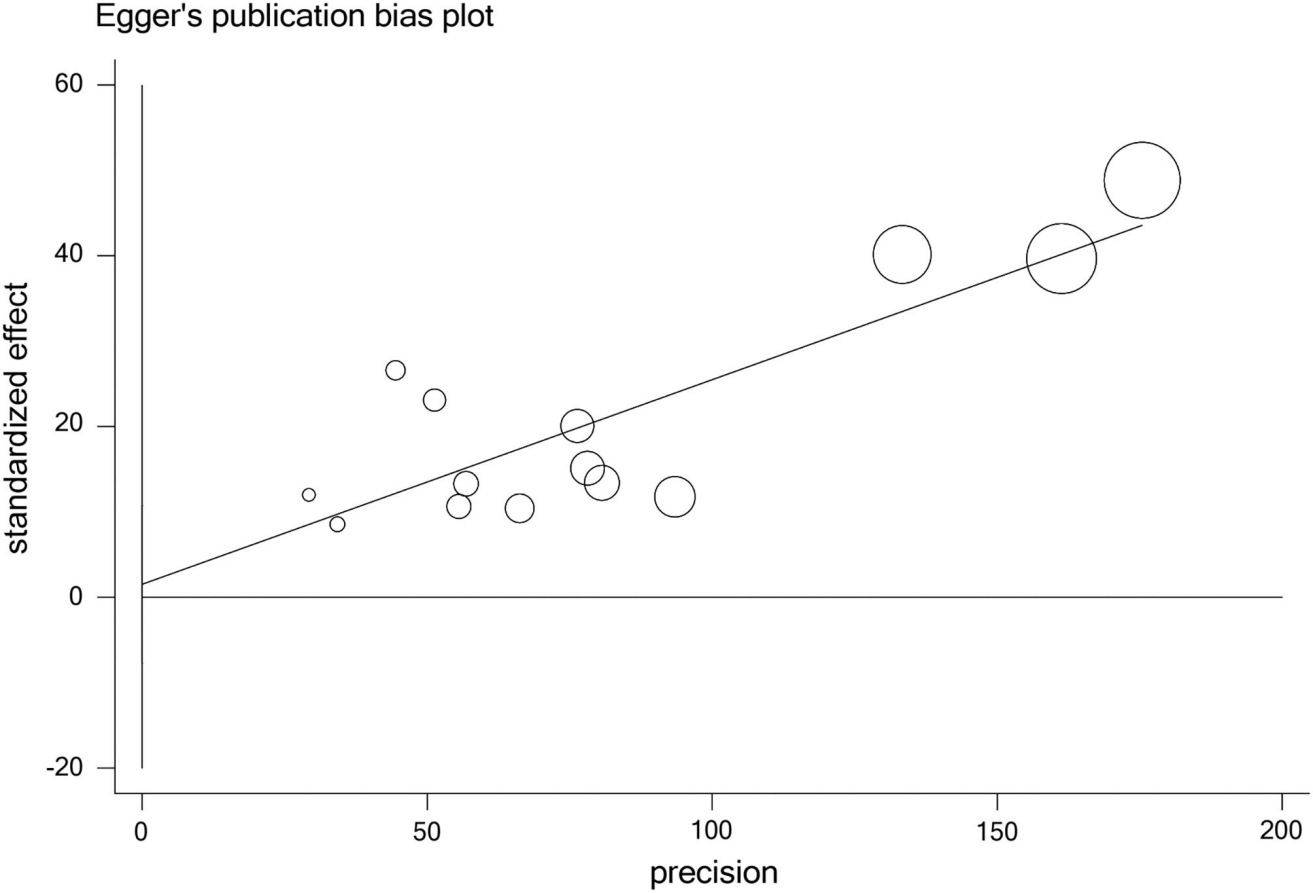
Bubble plots of publication bias.

The publication bias of studies was access by Begg's and Egger's tests. The results indicated no obvious publication bias in studies on sleep problems (*P*_Begg_ =0.228, *P*_Egger_ = 0.724) (Table 4, Figure 4).

**Table 4:** Publication bias.

	**Number of studies**	***P*-Value (95%CI)**
Begg' test	14	0.228
Egger' test		0.724 (−7.70, 10.76)

## Discussion

This study revealed that Chinese medical students have a prevalence of sleep problems (27.38%). Gender, region, education background, grade, and type of University were set as variables to conduct subgroup analysis. The results indicated that the prevalence in northern China was significantly higher than that in southern China. Besides, the results of the meta-regression indicated that the region was the significant heterogeneous source. The unique characteristics of regions in the north, such as higher latitudes which leads to different geographical attributes (42) and work-rest patterns, and limitation of the economic development level (43), may cause the higher prevalence of low sleep quality (44). We investigated the latitudes of the cities where these medical colleges are located, from the map website, and use GDP and GDP per capita as variables (Supplementary Table 2) reflecting the economic level of these cities (45, 46). The results of the meta-regression showed that latitude and GDP per capita are the sources of heterogeneity between studies, and the correlation analysis shows that prevalence is positively correlated with latitude (CC = 0.929, *P* <0.001) and negatively correlated with GDP per capita (CC = −0.919, *P* = 0.003), which consistent with the results of meta-regression. These results indicate that a high prevalence of sleep problems may occur in regions at a high latitude with a relatively low socioeconomic status.

The subgroup analysis also showed that the prevalence was higher in Western medicine universities than in TCM universities, and higher in undergraduates than in postgraduates. The prevalence of sleep problems in Grade 1 was slightly higher than that in Grade 2 and increased with the rising in grade from Grade 2. This may result from the Chinese medical education system has several unique characteristics. For example, clinical medical education has a variety of degrees and educational systems, including 5-year undergraduate and 3-year in both master's and doctor's programs (47). Therefore, the diversity of the medical education environment in China may lead to heterogeneity in this study. During the 5-year undergraduate education, medical students are exposed to medical knowledge from scratch, and the proportion of the course time is very high. Students in the first academic year face great challenges, resulting in a high prevalence of sleep problems. In the next few years, they also bear more profound medical knowledge and face a complex clinical practice. The results of the subgroup analysis showed that the prevalence of sleep problems followed an obvious upward trend from the second semester and reached the highest in the graduation year. This might be the reason why 60% of grade 5 undergraduates had sleep problems.

Previous studies reported that students major in TCM had better sleep than that with other majors (48). In this review, the prevalence of sleep problems in TCM universities is significantly lower than that in Western medical universities, probably because TCM students are better at adjusting life schedules according to their specialized courses. The courses in TCM universities involve acupuncture, massage, Qigong (49), Tai Chi (50), Chinese herbs, and so forth. TCM students practice iteratively for training, and they often apply these treatments to each other to help solve physical problems. These measures are effective in relaxing the body and improving sleep quality, which may have a positive impact on the low incidence of sleep problems among TCM students.

In this meta-analysis, PSQI was the main scale for detecting sleep quality, and only two studies applied other measurements. The heterogeneity caused by different measurements should be taken into account between studies. However, in the results of meta-regression, the heterogeneity between studies was not significant with different measurements (*P* = 0.689). No publication bias was found with Egger's and Begg's tests.

The prevalence of sleep problems at COVID-19 pandemic is also our research target. We searched several papers about sleep problems of Chinese adolescents during COVID-19 pandemic, but no study about medical students. Previous studies showed higher prevalence that 40–44.2% of children and adolescents have sleep disorders (51, 52). 39.7–60% of healthcare workers endured poor sleep quality and most of that are combined with moderate to severe stress during the outbreak of COVID-19 (51, 53). Medical students are similar to these populations and may be at a higher risk for sleep problems during the COVID-19 pandemic, which could be a suitable objective for future researches.

Medical students and related educational institutions in areas with high prevalence rate of sleep disorders require more attention on sleep problems. Regular monitoring is needed for better managements. Future researches could set subgroups and focus on the different regional characteristics and educational background, explore the pattern of the increasing prevalence of sleep problems of medical students year by year, and whether traditional Chinese medicine treatment has an impact on sleep disorders.

### Limitations

(1) The between-study heterogeneity in this review were significant. Except the variables already included, there may be other factors leading to heterogeneity. Several subgroups included only one study each on graduate students, fifth-grade undergraduates, and TCM students, leading to potential bias. (2) All included studies are cross-sectional designed, and therefore the longtime prevalence for a period cannot be observed. Also, a control group of healthy people was lacking in the design of each study. (3) The prevalence were assessed by self-report measurements and represent specific aspects of sleep problems. Objective and standard assessment is needed for future researches. (4) Further, the cut-off lines of PSQI in studies were not the same. There are several common criteria for judging low sleep quality with PSQI, score > 5 (54), > 6 (55), and > 7 (56, 57). The PSQI cutoff value of studies included for assessment in this review are all over 7 or 8, and that may cause underestimated prevalence compared to studies using PSQI with a cutoff of 5 or 6. (5) The evidence that meets the inclusion criteria during COVID-19 were lacking. (6) We chose GDP data in 2019 to reflects the economic level of cities, but these levels were at the same year, which may cause bias in range of years.

Hence, caution was required in interpreting the results. Besides, the AHRQ evaluation results showed that the proportion of high-quality studies included in this analysis was not high, only three high-quality studies. Hence, more attention should be paid to these issues in future research, and more hierarchical researches with subgroup analysis of region difference and education background should be conducted to investigate sleep problems more clearly and comprehensively.

## Conclusions

The prevalence of sleep problems among Chinese medical students is 27.38%. The region, educational background, grade, and University type may cause different prevalence. The difference in latitude between regions and level of socioeconomic status may cause differences in prevalence between studies. However, the results of the meta-analysis in this review should be explained with caution due to heterogeneity and potential bias among studies. This analysis emphasized the necessity of regular screening and appropriate intervention of sleep problems in Chinese medical students, and provide systematic evidence to improve medical education.

## Data Availability Statement

The original contributions presented in the study are included in the article/Supplementary Material, further inquiries can be directed to the corresponding authors.

## Author Contributions

YS, XW, and TJ proposed the meta-analysis and designed the study. YS, FQ, and TJ performed the systematic search and publication review. YS and HW evaluated articles for eligibility. YS, XW, and TJ performed data extraction, interpreted the results, and analysis. YS and TJ wrote the manuscript and revised all versions of the manuscript. XW and FQ conceived the study and critically reviewed the manuscript. All authors contributed to the article and approved the submitted version.

## Funding

This work was financially supported by the Shanghai Key Clinical Specialty Project (shslczdzk05301), National Natural Science Foundation of China (Grant Nos. 82074222 and 81573647), Guided project of Shanghai Science Technology Commission (19401934300), and Shanghai Key Laboratory of Traditional Chinese Clinical Medicine (14DZ2273200).

## Conflict of Interest

The authors declare that the research was conducted in the absence of any commercial or financial relationships that could be construed as a potential conflict of interest.

## Publisher's Note

All claims expressed in this article are solely those of the authors and do not necessarily represent those of their affiliated organizations, or those of the publisher, the editors and the reviewers. Any product that may be evaluated in this article, or claim that may be made by its manufacturer, is not guaranteed or endorsed by the publisher.
